# Literature-based considerations regarding organizing and performing cardiac surgery against the backdrop of the coronavirus pandemic

**DOI:** 10.1186/s13019-021-01419-9

**Published:** 2021-04-09

**Authors:** Andrzej Juraszek, Jarosław Kuriata, Piotr Kołsut, Tomasz Hryniewiecki, Monika Różewicz-Juraszek, Tomasz Dziodzio, Mariusz Kuśmierczyk

**Affiliations:** 1 Department of Cardiac Surgery and Transplantation, The Cardinal Stefan Wyszyński National Institute of Cardiology, Alpejska 42, 04-628 Warszawa, Poland; 2 Department of Valvular Heart Diseases, The Cardinal Stefan Wyszyński National Institute of Cardiology, Warsaw, Poland; 3 Department of Surgery, Campus Charité-Mitte and Campus Virchow-Klinikum, Charité –Universitätsmedizin Berlin, corporate member of Freie Universität Berlin, Humboldt-Universität zu Berlin, and Berlin Institute of Health, Berlin, Germany

**Keywords:** Cardiac surgery, Coronavirus disease 2019, Recommendations

## Abstract

**Background:**

The ongoing coronavirus disease 2019 (Covid-19) pandemic presents challenges for surgeons of all disciplines, including cardiologists. The volume of cardiac surgery cases has to comply with the mandatory constraints of healthcare capacities. The treatment of Covid-19-positive patients must also be considered. Unfortunately, no scientific evidence is available on this issue. Therefore, this study aimed to offer some consensus-based considerations, derived from available scientific papers, regarding the organization and performance of cardiac surgery against the backdrop of the Covid-19 pandemic.

**Methods:**

Key recommendations were extracted from recent literature concerning cardiac surgery.

RESULTSː Reducing elective cardiac procedures should be based on frequent clinical assessment of patients on the waiting list (every one or two weeks) and the current local status of the Covid-19 pandemic. Screening tests at admission for every patient are broadly recommended. Where appropriate, alternative treatment methods can be considered, including percutaneous techniques and minimally invasive surgery, if performed by experienced cardiac surgery teams.

**Conclusions:**

There is little evidence on the strategies to organize cardiac surgery in the Covid-19 pandemic. Most authors agree on reducing elective operations based on patients’ clinical condition and the status of the Covid-19 pandemic. Admission screenings and the use of percutaneous or minimally invasive approaches should be preferred to reduce in-hospital stays.

## Background

Global public healthcare providers are currently facing an unprecedented challenge in dealing with the coronavirus disease 2019 (Covid-19) pandemic. Readjustments in the field of cardiac surgery are critically important for several reasons: all phases of cardiac surgery (preoperative care, the surgery itself, postoperative care, and follow-up visits) require high capacities of trained staff, operating rooms, and — crucially during the pandemic — intensive care resources. In apportioning intensive care and staff capacities, all involved sectors are forced to triage time-sensitive interventions from those that can be safely delayed, or in some cases, substituted by less complex therapy options [1, 2]. However, in practice, there is a wide degree of medical urgency, ranging from elective operations to high emergency options. Therefore, the reasonable execution of a heart surgery programs affected by capacity shortage during the pandemic demands complex consideration and decision-making.

Given the high-speed dynamic of the Covid-19 outbreak, no scientific evidence is currently available to guide medical policymakers during this crucial period of readjustment. Therefore, clear guidelines and recommendations are urgently needed.

In this literature review, we aimed to identify the best practices to manage surgical programs until reliable scientific data are available.

## Methods

We searched PubMed for scientific articles using the following terms: “cardiac surgery” AND “coronavirus” OR “covid-19” or “heart surgery” AND “coronavirus” OR “covid-19”. The last search was performed on the 19th of January 2021 [3].

Identified manuscripts were then analyzed for available information on recommendations for cardiac surgeons during the Covid-19 pandemic. Proposed and discussed recommendations were pooled. The analysis did not require the use of any statistical methods. The acquired information was summarized, and key recommendations were generated.

## Results

A total of 20 publications were identified containing recommendations on the handling of cardiac surgery during the Covid-19 pandemic. The recommendations can be summarized as follows:

### General recommendations

>Postponing of elective operations should be based on patients’ individual risk [4–13].

>Triage and surgical indications should be adapted according to the local prevalence of Covid-19 and the hospital’s resources [4–6, 12].

>A hub-and-spoke organization system with dedicated cardiac centers may be applied in the acute phase of the epidemic [6, 7].

>The estimated time of the reduced capacity of cardiac surgery is not known [4–9].

>Each hospital should prepare its own guidelines to suit the restrictions based on the local situation [4–7].

Indeed, the incidence of Covid-19 vastly differs around the world, with various epicenters [1]. Therefore, we must achieve a good balance between the surgical treatment of thoracic pathologies and the preservation of hospital resources for Covid-19 patients. For example, in Lombardy, four reference and enrolment hub hospitals were identified [7]. Other hospitals, the spokes, were almost entirely treating Covid-19 patients, and became peripheral referral centers. Three different pathways were created: one for Covid-19-negative patients, one for patients awaiting screening results, and one for Covid-19-positive patients. The postoperative intensive care unit was split into two areas to create physical separation between Covid-19-positive and negative patients [7]. In contrast, Haft et al. [4] proposed a tiered patient triage guidance statement, as shown in Table 1. A similar triage and prioritization system was published by the Saudi Society for Cardiac Surgeons [5]. Meanwhile, Hussain et al. [6] suggested a service system for providing urgent and emergency cardiac surgery for all of London, focusing on maintaining a Covid-free in-hospital environment.

**Table 1:** The triage system during the coravirus-19 pandemic, as proposed by Haft et al. [4]. Covid-19- coronavirus disease 2019

***Essential services***	***Deferred***
**Tier 1 (0–30% inpatient Covid-19 load, mild reduction in operative capacity)**	
All in-patients waiting for surgery, including emergency services	Asymptomatic outpatients
Outpatients who are at greatest risk of adverse events	Truly elective interventions
	Asymptomatic or minimally symptomatic
	Severe mitral regurgitation
	Atrial septal defect and/or patent foramen ovale surgery
	Asymptomatic aneurysm with demonstrated stable size
	Isolated arrhythmia procedures
**Tier 2 (30–60% inpatient Covid-19 load, moderate reduction in operative capacity)**	
All in-patients waiting for surgery, including emergency services	Asymptomatic outpatients and patients with anatomy and physiology suggesting delay can be provided with reasonable safety
Outpatients with progressive symptomatology who have demonstrated failure of medical management	
Symptomatic coronary artery disease	
Asymptomatic coronary artery disease with impaired systolic function	
**Tier 3 (60–80% inpatient Covid-19 load, severe reduction in operative capacity)**	
All in-patients who cannot be discharged safely without surgical intervention/correction, including emergency services	All patients who are outpatients
	Patients deteriorating while waiting would need to meet criteria for admission before consideration for surgery
**Tier 4 (> 80% inpatient Covid-19 load, minimal operative capacity)**	
Only emergency services based on resource availability	All inpatients judged to be stable and capable of waiting
	All outpatients

Based on epicenter experience following clinical strategies have been recently proposed by George at al [14].: Patients with stable coronary disease are at relatively low risk of mortality and should be postponed. A similar strategy should be employed for valve operations. Patients with asymptomatic severe aortic stenosis should be postponed due to low annual risk of sudden cardiac death.

Left-sided endocarditis with hemodynamic compromise or very large vegetations should be prioritized over cases that can be managed medically until resources improve. Most patients with mitral valve pathologies can also be postponed but should be aggressively managed medically and monitored closely for signs of decompensated heart failure.

Patients with giant aneurysms (> 7 cm) as well as (saccular) pseudoaneurysms may be considered for urgent surgery depending on the risk of aneurysm-related death and the availability of resources. Asymptomatic patients with smaller aneurysms can have surgery delayed for 2 to 3 months. All in-patients, who cannot be discharged safely without surgical intervention (ascending aortic dissections, acute coronary syndromes, acute valvular endocarditis, and heart failure patients awaiting heart transplant or mechanical circulatory support) need completion of treatment [14].

>Follow up and reevaluation of waiting elective patients should be performed every 1–2 weeks [4].

Only regular contact with pending patients can guarantee their safety. The pandemic situation may also cause a fear of surgery, even in patients with symptomatic heart disease. High-risk patients should be prioritized to preventive strategies, including optimal antithrombotic management, medication review, and stringent monitoring [15]. The Heart Team making decisions about the treatment time should consist of cardiac surgeons, cardiologists, anaesthesiologists, radiologists and psychologists. Such a wide group of specialists guarantees the selection of the appropriate treatment time for patients who need to undergo cardiac surgery. The Heart Team should receive the Covid-19 specific recommendations [16].

>Local extracorporeal membrane oxygenation (ECMO) protocols for both Covid-19 and non-Covid-19 patients should be developed [4, 5, 10, 12].

Although most patients with Covid-19 develop moderate symptoms and recover quickly, some develop severe respiratory failure and acute respiratory distress syndrome, requiring intensive medical care. High mortality is observed in patients with Covid-19 requiring mechanical ventilation. ECMO can save lives in patients with severe respiratory distress syndrome or refractory treatment of heart failure. Primarily, ECMO is widely used in cardiac surgery to treat acute cardiac failure and postcardiotomy cardiogenic shock.

> Left ventricular assist device implantation should not be restricted as the treatment of choice for advanced heart failure due to the lack of healthy donor hearts for cardiac transplantation [17].

### Staff recommendations

>Staffing should be kept to a minimum [4, 2].

>Telemedicine techniques should be widely used [4, 9, 10].

>Home office strategy should be applied where possible [4, 9].

>Surgical masks should be worn at any patient contact [4, 11].

The purpose of the above recommendations is to reduce the risk of infection occurrence and spread among hospital staff.

### Patient screening

>Travel to the hospital by patients and accompanying relatives should be limited [4–9].

>In-person clinic evaluations should be appraised [4–9].

>All patients should be evaluated for respiratory symptoms before hospitalization [4, 11].

>Nasal swab screening for Covid-19 has to be done the same day of admission to the hospital [4, 8, 11].

>Elective or semi-elective cardiac surgeries should be postponed until Covid-19 virus detection results are negative [11].

>All patients should wear surgical masks [4, 9, 11].

Patient recommendations are intended to ensure their safety and prevent a new source of infection in the hospital.

### Perioperative time

>A designated theatre and scrub room should be used for suspected or proven Covid-19 patients [10–12].

>A preoperative Covid-19 checklist should be used for suspected and confirmed Covid-19 patients [11].

>The number of personnel should be kept to a minimum [8–10, 12].

>Appropriate personal protective equipment (i.e., ≥PPE2) should be used for all patients, and in the case of Covid-19-positive patients, PPE2/3 and goggles should be worn [10–12].

General precautions are therefore recommended to prevent the spread of Covid-19 infection through isolation, protective measures, and staff reduction. Patients should be transferred directly to the operating room without stopping in the intesive care unit areas to minimize exposure for patients and staff [18].

>Performing minimally invasive operations in experienced centers may shorten the hospital stay and usage of blood products [19].

>Preference for percutaneous techniques instead of surgery (i.e., coronary interventions, aortic valve replacement, transcatheter aortic valve implantation (TAVI)) [11, 12].

>Extensive use of strategies to avoid wound infections after surgeries [20].

Changing the surgical strategy can be beneficial for experienced teams.

Based on the available literature, the above recommendations can be summarized as follows:

1. Selectively reduce elective cardiac surgery procedures based on continuous assessment of patients on the waiting list (every one or two weeks) and the current state of the epidemic.
2. Perform time-sensitive operations without restrictions.
3. Limit the presence of staff in the clinic and maximize the use of telemedicine.
4. Perform screening tests on patients admitted to the clinic, and await results before undertaking further action.
5. Use percutaneous techniques and minimally invasive procedures if experienced hospital staff are available (to provide benefit and reduce risk).
6. Develop local protocols for patients with suspected or diagnosed Covid-19.
7. Provide protective means including surgical masks for all hospital staff and patients (appropriate protection is defined as ≥ PPE2 for all patients and hospital staff, and PPE2/3 and goggles for Covid-19-positive patients).
8. Participate in the treatment of Covid-19 patients using ECMO provided there are sufficient resources and experienced personnel to do so.

## Discussion

Due to the unexpected challenges of the Covid-19 pandemic, typical scientific guidelines for cardiac surgery are not available. Available publications usually reflect the opinion and experience of individual authors.

The reduction or cancellation of elective heart surgery was identified as the main topic of most publications. Micelli et al. reported their experience in April 2020 and recommended the limitation of procedures to urgent operations only in the region of Lombardy [21]. This recommendation aims to focus all efforts on combating Covid-19 at the start of the pandemic [4]. Some authors also recommended applying a hub-and-spoke system with dedicated cardiac centers [6, 7]. However, the epidemic is now entering a chronic phase in many countries, and it is not known how long this state will last. Therefore, during this chronic period, most authors suggest performing a limited number of elective procedures based on a triage system [4, 5]. Deferring patients already on a waiting list generates anxiety and may also provoke the onset of cardiac and non- cardiac symptoms like hypertensive crisis, sleep disorders. The best solution for these patients is the increased care of cardiologists and family doctors. Since access to them may be temporarily limited, the use of telemedicine techniques should be extended in this field. In addition, access to a psychologist in a telemedical mode should be guaranteed. Additionally, telemedicine can be used to assess patient’s symptoms and condition and is becoming more important during the Covid-19 pandemic, as it prevents physical contact and reduces the need for patients and their relatives to access healthcare facilities [8–10]. Telemedicine can also reduce the number of staff present in the hospital. Indeed, many authors suggest keeping the staff present in the hospital to the necessary minimum. Staff working from home can also conduct documentary, statistical, and scientific activities [6–8]. Furthermore, in many cases, the treatment strategy for a given patient can be decided before hospital admission, and telemedicine can be useful for referring patients for echocardiography or cardiac angiography examinations.

Another recommendation is to favor percutaneous techniques, such as coronary stenting and TAVI [11, 12]. In addition, an expert consensus stated that minimally invasive procedures could minimize the need for blood products, reduce hospital stay, and shorten rehabilitation time [11]. Meanwhile, electrosurgical and ultrasonic devices, as well as CO_2_ insufflation should be used with care to avoid the risk of aerosolization [19]. However, to date, there is no evidence of the pros and cons of less invasive techniques during the Covid-19 pandemic. Nonetheless, it is advised that blood products should be largely reserved for patients being treated for Covid-19. Additionally, whether Covid-19 can be transmitted by blood transfusion remains unclear [22]. Finally, there has been a reduction in blood donation rates during the pandemic, which should be considered when planning a procedure [9].

It is currently unclear whether the training of residents during the Covid-19 pandemic should continue. Stopping education in the acute phase of a pandemic is understandable, yet a break in training lasting several months would be not recommended. Therefore, the current recommendation is to minimize exposure among trainees and students, which would also reduce PPE usage (as access is still limited) [9].

Patients admitted to the cardiac surgery department should routinely undergo screening for Covid-19, and the results should be obtained before further action is undertaken. Such action is required as the admission of Covid-19-positive patients increases the probability of Severe acute respiratory syndrome (SARS) coronavirus transmission to staff members, and quarantining such staff would entail excluding a significant proportion of the workforce. Interestingly, systematic polymerase chain reaction-based (PCR) testing was not broadly recommended by all authors [10–12]. If a patient does test positive for Covid-19, PPE should be provided to all staff caring for that patient. Appropriate protection was defined as ≥PPE2 for all patients and hospital staff, with PPE2/3 and goggles required in the case of Covid-19-positive patients [10–12].

Gaudino et al. [23] quantified the experience and changes implemented in response to the Covid-19 pandemic across cardiac surgery centers participating in an international research consortium. Out of 61 centers, 98.3% completed the worldwide survey (19 countries). The median reduction in surgery case volume was 50–75%, and strictly correlated with the number of local Covid-19 cases. A third of the centers reported a > 50% reduction in the number of cardiac operating rooms and intensive care unit beds. Most centers restricted their activity to urgent or emergent cases. Interestingly, 5% of centers had canceled all operations, including emergencies [23]. Czerny at el. found that was no change in the number of acute thoracic and abdominal aortic cases and procedures during the initial wave of the Covid-19 pandemic, whereas the case load of elective operations and procedures decreased significantly. Covid-19 positive patients with acute aortic syndromes were managed and operated to current guidelines [24].

Between March 16, 2020 and May 15, 2020, a total of 54 adult patients underwent cardiac surgery at Weill Cornell Medicine in New York beeing an epicenter of pandemics. There was one operative mortality (1.9%) associated with an acute perioperative Covid-19 infection [25].

The Covid-19 pandemic is a new phenomenon in global healthcare. Currently, it is difficult to expect recommendations based on scientific evidence; however, in time, it will become possible to collect data on the results of cardiac surgery during a pandemic. Such data should include mortality rates among those on waitlists, the outcomes of operations, and the number of Covid-19-related deaths following cardiac surgery. Current data suggests an increase in operative mortality in waitlist patients [26]. However, the degree to which resources have shifted from cardiac surgery to treatment of Covid-19 patients is unclear.

## Conclusions

To conclude, the majority of the authors reported reduced cardiac surgery activity, redeployed personnel, and curtailed educational and research activities in response to the Covid-19 pandemic. The volume reduction in cardiac surgery may not lead to enhanced non-Covid-19 mortality.

## Data Availability

The datasets used and/or analysed during the current study are available from the corresponding author on reasonable request.

## Abbreviations

Covid-19: Coronavirus disease 2019
PPE: Personal protective equipment
TAVI: Transcatheter aortic valve implantation
PCR: Polymerase chain reaction
ECMO: Extracorporeal membrane oxygenation
